# The Functional Role of Lipoproteins in Atherosclerosis: Novel Directions for Diagnosis and Targeting Therapy

**DOI:** 10.14336/AD.2021.0929

**Published:** 2022-04-01

**Authors:** Yongzheng Lu, Xiaolin Cui, Li Zhang, Xu Wang, Yanyan Xu, Zhen Qin, Gangqiong Liu, Qiguang Wang, Kang Tian, Khoon S Lim, Chris J Charles, Jinying Zhang, Junnan Tang

**Affiliations:** ^1^Department of Cardiology, the First Affiliated Hospital of Zhengzhou University, Zhengzhou, Henan, China.; ^2^Key Laboratory of Cardiac Injury and Repair of Henan Province, Zhengzhou, Henan, China.; ^3^Henan Province Clinical Research Center for Cardiovascular Diseases, Zhengzhou, Henan, China.; ^4^Christchurch Regenerative Medicine and Tissue Engineering (CReaTE) group, Department of Orthopedic Surgery, University of Otago, Christchurch 8011, New Zealand.; ^5^Department of Bone and Joint, the First Affiliated Hospital of Dalian Medical University, Dalian, Liaoning, China.; ^6^Department of Medical Record Management, the First Affiliated Hospital of Zhengzhou University, Zhengzhou, Henan, China.; ^7^National Engineering Research Centre for Biomaterials, Sichuan University, Chengdu, Sichuan, China.; ^8^Christchurch Heart Institute, Department of Medicine, University of Otago Christchurch, Christchurch 8011, New Zealand

**Keywords:** atherosclerosis, dyslipidemia, lipoproteins, HDL, LDL

## Abstract

Dyslipidemia, characterized by a high level of lipids (cholesterol, triglycerides, or both), can increase the risk of developing and progressing atherosclerosis. As atherosclerosis progresses, the number and severity of aterial plagues increases with greater risk of myocardial infarction, a major contributor to cardiovascular mortality. Atherosclerosis progresses in four phases, namely endothelial dysfunction, fatty streak formation, lesion progression and plaque rupture, and eventually thrombosis and arterial obstruction. With greater understanding of the pathological processes underlying atherosclerosis, researchers have identified that lipoproteins play a significant role in the development of atherosclerosis. In particular, apolipoprotein B (apoB)-containing lipoproteins have been shown to associate with atherosclerosis. Oxidized low-density lipoproteins (ox-LDLs) also contribute to the progression of atherosclerosis whereas high-density lipoproteins (HDL) contribute to the removal of cholesterol from macrophages thereby inhibiting the formation of foam cells. Given these known associations, lipoproteins may have potential as biomarkers for predicting risk associated with atherosclerotic plaques or may be targets as novel therapeutic agents. As such, the rapid development of drugs targeting lipoprotein metabolism may lead to novel treatments for atherosclerosis. A comprehensive review of lipoprotein function and their role in atherosclerosis, along with the latest development of lipoprotein targeted treatment, is timely. This review focuses on the functions of different lipoproteins and their involvement in atherosclerosis. Further, diagnostic and therapeutic potential are highlighted giving insight into novel lipoprotein-targetted approaches to treat atherosclerosis.

## 1. Introduction

Atherosclerosis is one of the most common causes of cardiovascular disease (CVD), leading to about 7.2 million deaths each year [1], and the American Heart Association has reported that the prevalence of atherosclerosis will increase by 18% by 2030 [1]. Risk factors for atherosclerosis include dyslipidemia, hypertension, obesity, smoking, diabetes, abnormal glucose tolerance, age, gender, and family history. Of these, recent research has suggested that dyslipidemia is one of the main risk factors contributing to the incidence and progression of atherosclerosis. Dyslipidemia is a common condition characterized by high plasma levels of cholesterol, triglycerides, or both [2] and measurement of plasma cholesterol and triglycerides levels are reliable diagnostic markers of dyslipidemia. Triglycerides and cholesterol are major lipids in the blood that are transported by lipoproteins and have different physiological functions. Triglycerides are a key source of calories (energy) throughout the body and also store excess heat. Cholesterol is an essential structural constituent of cell membranes and is an essential constituent of some hormones (e.g., steroid hormones). In healthy people, when levels of glycerides/cholesterol increase within the blood, cells such as hepatocytes secrete lipoproteins that play an essential role in lipid transport, metabolism, and storage. Binding of lipids to the lipoproteins facilitates a rebalance of lipid metabolism and returns circulating levels of unbound glycerides/cholesterol to more normal levels. Lipoproteins are classified into five categories based on their density and size, namely very-low-density lipoprotein (VLDLs), low-density lipoproteins (LDLs), intermediate-density lipoproteins (IDLs), high-density lipoproteins (HDLs), and chylomicrons (CM). The various lipoproteins bind different proportions of triglycerides and cholesterol and are associated with different types of apolipoproteins [3]. The unique composition of each lipoprotein determines its specific function in maintaining the balance of lipid metabolism [4]. The various lipoproteins work together cohesively to maintain healthy vasculature, including the coroanary arteries. However, with prolonged dyslipidemia, the lipoproteins fail to maintain healthy lipid metabolism, resulting in sustained high levels of cholesterol and triglycerides and accelerated accumulation of lipids in atherosclerotic plaque, thereby exacerbating atherosclerosis.

**Figure 1.**
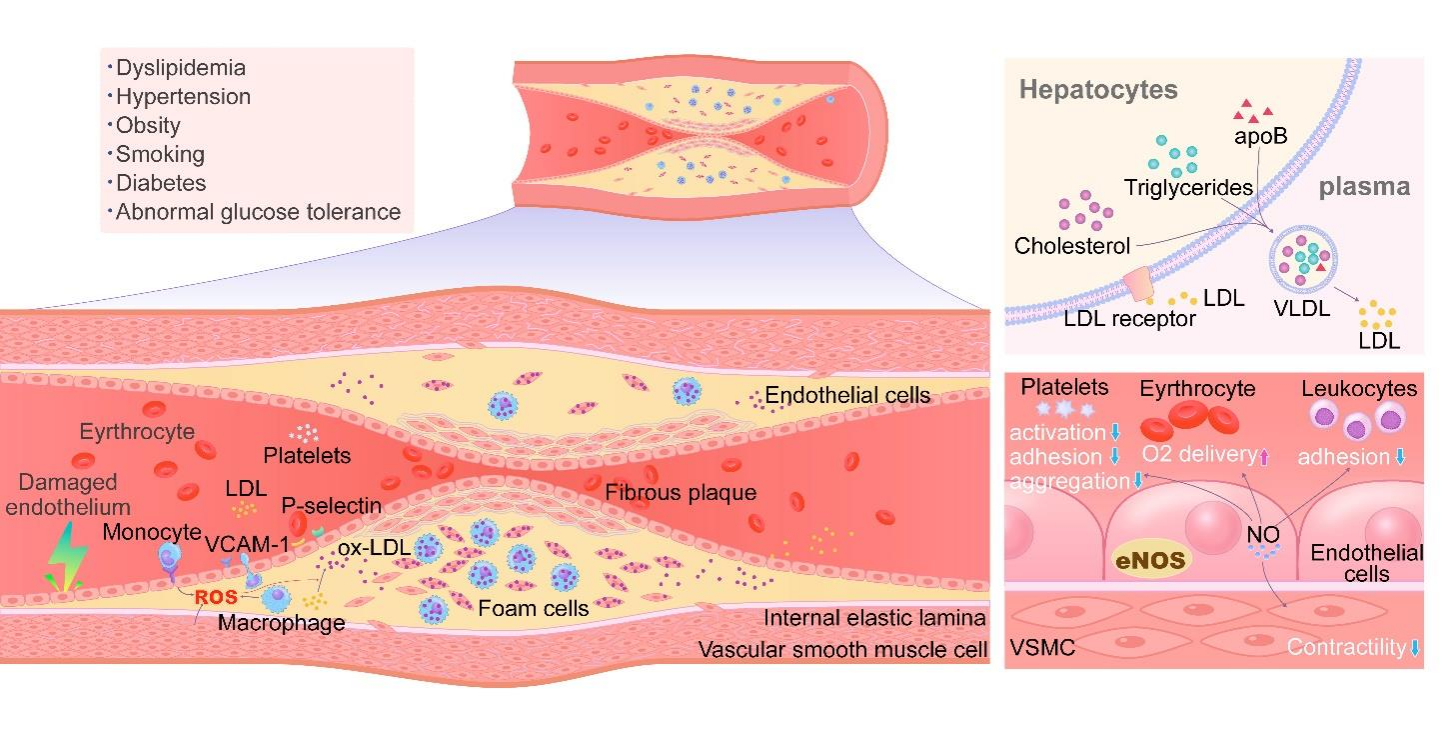
Summary of the mechanism of atherogenesis. Triglycerides, cholesterols and apoB comprise VLDLs in liver, which are secreted into the circulation. Most of the triglycerides are removed by lipoprotein lipase leading to the VLDLs’ transformation into LDLs. In normal metabolism, LDLs are removed from circulation via LDL receptors on the surface of hepatocytes. However, when the excessive secretion of lipoproteins by the liver and/or ineffective clearance of plasma LDLs occurs, the level of plasma LDL elevates. Furthermore, various risk factors (including dyslipidemia, hyper-tension, obesity, smoking, diabetes, and abnormal glucose tolerance, etc.) stimulates endothelial cells resulting in endothelial damage. Compared with intact endothelium, NO production is deficient in impaired endothelial, which induces platelet aggregation, endothelial-leukocyte interactions and thrombosis. Moreover, damaged endothelium has increased permeability for lipid particles, which accelerates lipid deposition in the sub-intima. Damaged vascular endothelial cells could express VCAM-1, ICAM-1, MCP-1 and IP-10, which attract monocytes and lymphocytes, and leads to the consequential infiltration into the sub-intimal space. Simultaneously, SMCs derived from the arterial media layers also migrate into the sub-intimal space through the membrane pores in the internal elastic lamina. All of these accelerate the formation of foam cells and the process of atherosclerotic.

Multiple steps are involved in the pathogenesis of atherosclerosis, including lipid infiltration, endothelial damage, inflammatory response, platelet aggregation, and thrombosis (Fig. 1). Endothelial damage is considered the first step preceding the formation of atherosclerotic plaque. A key function of endothelial cells is to prevent adhesion in the vasculature. Endothelial dysfunction is induced by conditions such as hypertension, metabolic syndrome, smoking and physical inactivity, resulting in senescence and apoptosis of endothelial cells [5]. Abnormal or damaged endothelium increases expression of inflammatory factors like adhesion molecules with enhanced permeability, resulting in increased numbers of lipid particles penetrating the intima and sequential accumulation of lipid within the sub-intima. Lipoproteins continue to enter into the subintimal space and subendothelium, either through transcytosis, or via gap junctions and other processes [5]. Infiltration of lipoproteins, particularly LDL, contributes to the development of atherosclerosis. LDL often enters and accumulates in subendothelium of the vascular wall via transcytosis (promoted by inflammatory factors such as tumour necrosis factor α (TNF-α)). LDL then undergoes oxidative modification induced by vascular smooth muscle cells (SMCs), monocytes, and endothelial cells, resulting in the formation of oxidized LDL (ox-LDL) [6]. Ox-LDL further aggravates endothelial damage and stimulates endothelial cells to produce molecules such as E-selectin or P-selectin, promoting the migration of circulating monocytes towards the vascular wall [7]. Ox-LDL stimulation of endothelium also induces expression of monocyte chemoattractant protein-1 (MCP-1) resulting in monocytes being attracted and traversing the endothelial intercellular gap, entering the sub-intimal space of the vascular wall and accumulating at the lesion site [8]. Similarly, TNF-α stimulates endothelial cells to express interferon-gamma-induced protein 10 (IP-10) which then participates in the collection and migration of lymphocytes from blood into the arterial wall. Both vascular cell adhesion molecule-1 (VCAM-1) and intercellular adhesion molecule-1 (ICAM-1) are secreted by endothelial cells and facilitate capture of monocytes by damaged endothelium. Monocytes accumulating in the damaged endothelium proliferate further signals, promoting increased migration of monocytes to the lesion site resulting in further adhesion of monocytes and lymphocytes to endothelial cells with progressive migration into sub-intimal space, thereby facilitating a vicious cycle. Simultaneously, under the influence of mitogen-responsive nuclear factors, SMCs of the arterial media layers also migrate into the sub-intimal space through the fenestrated pores in the internal elastic lamina [9]. Monocytes transform into macrophages and, together with SMCs, take up ox-LDL by a variety of mechanisms, resulting in the formation of foam cells. Monocyte-derived macrophages engulf ox-LDL via a number of scavenger receptors (SRs) including CD36 receptors, and Fc receptors (FcRγs) which are implicated in the formation of monocyte-derived foam cells. Meanwhile, SMC-derived macrophages can engulf ox-LDL via lipoprotein lipase (LPL) receptors, leading to the formation of muscle-derived foam cells. Taken together, ox-LDL is the main contributor to the formation of foam cells, and foam cell formation is a key early event in atherosclerotic plaque development [3]. Interestingly, modified LDLs are exclusively captured by the macrophage scavenger receptor system, and ox-LDL production is not controlled by a feedback mechanism, thus resulting in an unlimited uptake [3]. The early atherosclerotic plaque then progresses to intimal thickening and narrowing of the vascular lumen, eventually resulting in vessel occlusion with thrombus formation with resultant clinical symptoms and adverse outcomes.

Understanding of functional role of lipoproteins in atherosclerosis has progressed in recent decades. In addition, new therapeutics have been developed and trialled targeting lipoproteins as treatment for atherosclerosis. Thus, a comprehensive review of lipoproteins’ function and their role in atherosclerosis, along with the latest development of lipoprotein targeted treatment, is timely. In this review, we summarise the different types of lipoproteins and their functions. We then outline the process of atherosclerosis development elucidating key links between lipoproteins and atherosclerosis. Lastly, we discuss the development of lipoprotein targeted treatment of atherosclerosis, including progress on novel delivery platforms for lipoprotein-based therapeutics. Together, this will provide insight to understanding new strategies for clinical management of atherosclerosis.

## 2. Apolipoprotein B (apoB)-containing lipoproteins in atherosclerosis

### 2.1 LDL

LDL particles are the main compartment storing serum lipids in the human body [10]. They are composed of free triacylglycerols, cholesterol, phospholipids, cholesteryl esters, and apoB [3], and act as the main transporters of cholesterol in human blood [3]. During normal metabolism, LDLs are removed from circulation via LDL receptors on the surface of hepatocytes [11]. However, when excessive secretion of lipoproteins by the liver and/or ineffective clearance of plasma LDL occurs, circulating levels of LDL rise [12]. Several studies have identified that raised plasma LDL levels results in the initiation and development of atherosclerotic plaques. This increases the risk of atherosclerosis-associated disease and contributes to the burden of cardiovascular disease [13-16]. Generally, a combination of VLDL, LDL, lipoprotein (a) and chylomicrons all contribute to the amount of cholesterol deposited in the arterial wall, and hence progression of atherogenesis. Of note, all of these particles contain apolipoprotein B (apoB). Overexpression of ARL15, a member of the Adenosine diphosphate-Ribosylation Factor (ARF) family, in the liver of mouse models has also been shown to lead to hypercholesterolemia via increasing apoB level. Hypercholesterolemia is a major risk factor contributing to atherosclerosis. Despite the fact that approximately 90% of apoB particles in circulation are resident in LDL particles [17], apoB is of increasing interest in atherosclerosis research, due to its ability to predict the atherogenic risk, which is more accurate than other markers such as LDL-C and HDL-C [18]. Mendelian randomized trials have revealed that the clinical benefits of cholesteryl ester transfer protein (CETP) inhibitors reflect the decrease in circulating LDL particles that are determined by apoB, rather than LDL-C [19, 20]. Another study showed that genetic variants mimicking LDL-C-lowering and triglyceride-lowering therapies play a role in decreasing atherosclerotic cardiovascular disease (ASCVD) risk for similar changes in apoB levels, despite the fact that plasma LDL-C and triglyceride levels showed different patterns of change [21]. These findings strongly suggest that ASCVD risk is correlated to the total apoB levels rather than lipid contents *per se*. As a result, the European Society of Cardiology/European Atherosclerosis Society 2019 guidelines for the management of dyslipidemia recommended measurement of apoB levels to estimate ASCVD risk and to assess the clinical benefits of using lipid-lowering therapy [22].

Aside from its role as a biomarker for diagnosis and prognosis of ASCVD risk, apoB may also have therapeutic potential for treatment of atherosclerosis. Lipid-lowering therapies (targeting LDL-cholesterol, HDL-cholesterol, total cholesterol, and triglycerides) are the current mainstream treatment paradigm for lowering ASCVD risk. Examples include strategies to upregulate LDL receptors, thus reducing LDL clearance and hence LDL-C, using agents such as ezetimibe, statins, and proprotein convertase subtilisin/kexin type 9 (PCSK9) inhibitors [11]. Such treatments are effective in many patients and can reduce major coronary event rates by ~30% [23]. However, because multiple pathological processes are in play in atherosclerosis, many patients still experience atherosclerosis complications despite successfully decreasing their cholesterol levels. Studies have shown that for some patients with elevated triglyceride levels and low LDL-C levels, therapy that targets management of apoB levels is a viable alternative. For example, John et al. demonstrated that antisense inhibition of apoB led to reductions of <50% apoB and <35% LDL-C levels in humans, suggesting targeting apoB as a potential new treatment strategy to reduce cardiovascular risk [24]. A recent study suggests that measurement of apoB should be included to guide the optimal treatment of patients [11]. ESC/EAS 2019 guidelines state that apoB analysis is recommended as secondary lipid analysis for risk evaluation, especially for patients who have high TG levels, diabetes mellitus, obesity, metabolic syndrome or very low LDL-C levels [22]. For patients at very-high, high and moderate CVD risk, the apoB level should be <65 mg/dL, <80 mg/dL and <100mg/dL respectively [25, 26]. It is also proposed that clinicians should consider the apoB lipoprotein lowering strategy to complement existing clinical practice for atherosclerosis treatment [11].

### 2.2 Triglyceride-rich lipoproteins

Multiple lines of evidence indicate that high levels of LDL and cholesterol contribute to atherosclerosis, whilst increased levels of HDL reduce the risk of developing atherosclerosis. However, there is no consensus on the atherogenic potential of elevated plasma triglycerides (TGs) and the triglyceride-rich lipoproteins (TRLs; including VLDL and chylomicrons) [27, 28].

TGs are transported in plasma by VLDLs, chylomicrons and their remnants created during metabolism [29]. Lipoprotein lipase (LPL) is a key tissue enzyme, expressed in heart, skeletal muscle and adipose tissue, that participates in the clearance of TRLs. LPL hydrolyzes TRLs in plasma, producing VLDL remnants (including chylomicron remnants and IDL) and high concentrations of lipolytic products such as oxidized free fatty acids [30]. Oxidized free fatty acids can increase expression of inflammatory cytokines and interleukins leading to endothelial inflammation [31-33]. There is some evidence that TRLs may be more atherogenic than LDL, and TRL cholesterol, rather than TGs, may directly promote atherosclerosis development [34, 35]. This may relate to larger volumes of TLR with chylomicrons (75-450 nm diameter) and VLDL (60-80 nm diameter) being the largest lipoprotein particles with greater capacity to carry more cholesterol than the smaller LDLs (18-25 nm diameter) [34].

TRLs and their remnants can upregulate the endothelial expression of ICAM-1 and VCAM-1 [36, 37], which facilitates endothelial monocyte adhesion and the transendothelial migration of leukocytes to the lesion site, leading to the exaggerated inflammatory responses [38]. They can also increase ROS production, resulting in cellular injury and death, and increase vascular endothelial permeability [33, 39]. Furthermore, TRLs and their remnants amplify the coagulation cascade by upregulating the expression of plasminogen activator inhibitor-1 and plasminogen activator inhibitor-1 antigen, while supporting the prothrombinase complex assembly, promoting platelet aggregation and thrombus formation [40]. TRL remnants can also accumulate on atheroprotective HDL particles [41], impairing their atheroprotective effect and impairing coronary vasodilation [42]. Taken together, TRLs and their remnants likely play key roles by multiple mechanisms to facilitate endothelial dysfunction thereby promoting atherogenesis.

### 2.3 A promising target: lipoprotein A

Lipoprotein a (Lp(a)), first discovered by Berg et al. (1963), is a circulating lipoprotein produced by the liver that is associated with atherosclerosis. Given the similarity of Lp(a) to LDL it may act in similar fashion to induce atherogenesis [43]. Lp(a) structure closely resembles a very large apo(a) protein linked via lysine pockets with apoB-100. Lp(a) promotes development of atherosclerosis by triggering endothelial dysfunction, inducing smooth muscle proliferation, and promoting macrophage foam cell formation [43]. A strong correlation has been reported between increased Lp(a) levels, particularly >50 mg/dL, and cardiovascular events including peripheral arterial disease, aortic stenosis, ischemic stroke, and myocardial infarction [44-48]. To date, no drugs targeting Lp(a) have been clinically approved. Statins have minimal effect on Lp(a) levels, and in fact Lp(a) levels increased in some patients. Similarly, nicotinic acid (niacin), estrogen, and PCSK9 inhibitors only reduce Lp(a) by small degrees (20% to 30%). With the estimated level required for significant reduction of Lp(a) being >50%, existing drugs fall to meet this threshold. There remains an opportunity to develop new drugs targeting Lp(a) that may meet this therapeutic threshold.

Despite increasing awareness of the importance of Lp(a), the measurement of Lp(a) still lacks standardization. Furthermore, specific mechanisms of action of Lp(a) in atherosclerosis remain elusive. Taken together, accumulating evidence indicates that Lp(a) could play a role as a biomarker in atherosclerosis diagnosis and may yet prove a novel therapeutic target.

## 3. Ox-LDL

### 3.1 The function of Ox-LDL in atherosclerosis

The natural course of atherosclerosis in both human and experimental animal models of hypercholesterolemia show that LDLs are highly susceptible to oxidative damage by oxygen radicals. LDL undergoes many changes in chemical composition after oxidation, including the generation of lipid peroxides and oxysterols, the degradation of apoB, and the transformation of LDL lipid components from phosphatidylcholine to lysophosphatidylcholine (lysoPC) [49, 50]. LysoPC, the lipid component of ox-LDL, is a significant intermediate product of LDL oxidation and the major active component of ox-LDL [51, 52]. LysoPC can enhance oxidative stress, induce inflammatory reactions, interfere with endothelial cell function and reduce plaque stability, possesses pro-atherosclerotic effect and participates in all stages of atherosclerosis. Under normal conditions, native LDL contains very low levels lysoPC with normal plasma levels being 12~166 μmol/L [53-55]. During oxidation of LDL, about 40% phosphatidylcholine can be transformed to lysoPC through two different pathways. The first and main route is activated secretory phospholipase A2 (sPLA2), an acute-phase protein, that can hydrolyze the fatty acid on the second carbon of LDL phosphatidylcholine, thereby making phosphatidyl-choline which transforms to lysoPC. Secondly, lecithin cholesterol acyltransferase (LCAT) can transfer fatty acid on the phosphatidylcholine, inducing related cholesterol and lysoPC formation. The emergence of oxidized LDL (ox-LDL) in atherosclerotic plaques serves as the starting point for the “oxidation hypothesis of atherosclerosis.” Taken together, ox-LDL is a collective term for heterogeneous oxidative changes in LDL lipid moieties [56].

High levels of ox-LDL are generally considered a high-risk factor for a cardiovascular events due to its central role in atherosclerotic plaque formation. Gao et al. (2017) reported that the incremental level of ox-LDL in the serum was correlated to the elevated risk of cardiovascular events in nested case-control studies, 10 case-cohort studies, and 11 prospective cohort studies on the relationship between ox-LDL and ASCVD [57]. Further studies have elucidated the role of ox-LDLs in the pathogenesis of atherosclerosis, especially their capability to promote inflammatory responses [49, 58, 59], inducing the loss of inherent antioxidant function in the body. For example, ox-LDLs trigger the activation of macrophages via Toll-like receptor-4 (TLR4) as well as accelerate reactive oxygen species (ROS) production and inflammatory cytokine oxidation [60].

Several studies have demonstrated that ox-LDLs can interact with multiple cell types (such as fibroblasts, SMCs, endothelial cells, macrophages and platelets,) and affect their normal physiological functions through various signalling pathways. For example, ox-LDLs can stimulate the abnormal proliferation, migration and collagen synthesis of vascular SMCs and fibroblasts via LOX-1, the receptor for ox-LDL, causing the fibrous hyperplastic pathological change of atherosclerotic arterial walls [61]. Furthermore, ox-LDL can stimulate endothelial cells to produce cytokines, including MCP-1, VCAM-1 and ICAM-1 and p-selectin. These cytokines can attract circulating monocytes in the blood to the damaged endothelium, which aggravates inflammatory cell infiltration of endothelial cells, further exacerbating endothelium damage. In normal physiological states, endothelium can take in L-arginine and produce a basal level of nitric oxide (NO) via a unique isoform of NO synthase (eNOS), catalyzing L-arginine and converting into L-citrulline and NO [62]. NO produced by endothelial cells can diffuse quickly via the endothelial plasma membrane to activate guanylate cyclase in several cell types present in the blood (such as platelets, leukocytes and SMCs). Activated guanylate cyclase in platelets can inhibit platelet activation, adhesion, and aggregation. Activated guanylate cyclase in leukocytes reduce their adhesion, and in SMCs can regulate vasorelaxation and dephosphorylation of the myosin light chain. Moreover, NO can also interact with hemoglobin in erythrocytes to enhance oxygen delivery to tissues [63]. However, ox-LDL can reduce the uptake of L-arginine by endothelial cells, thereby reducing the NO formation. Therefore, the vascular protective function of NO in injured endothelium is decreased to varying degrees or lost completely, which results in lipid deposition, inflammatory cell infiltration, platelet aggregation and thrombosis, accelerating the process of atherogenesis. Fei et al. showed that ox-LDL can induce NF-κB p65 phosphorylation and activate caspase 3, resulting in endothelial cell apoptosis [64]. Cao et al. showed that ox-LDL induces RAW264.7 macrophage-derived foam cell formation, promotes cell lipid accumulation, and induces a senescence phenotype with a reduced number of live cells [65].

Taken together, ox-LDLs interact with a variety of cells impacting normal physiological functions through multiple pathways, which promotes atherosclerosis development. As such, ox-LDLs are considered as key link in atherosclerotic plaque formation.

### 3.2 Novel direction for atherosclerosis diagnosis and treatment: targeting ox-LDL

Atherosclerotic plaques with high ox-LDL levels are at greater risk of rupture [66]. For this reason, ox-LDL may have potential as a marker of the “vulnerable plaque,” particularly relevant in targeted diagnostic imaging of atherosclerosis and in targeting delivery of therapeutic agents [56].

Coronary angiography visualizing radio-opaque contrast injected into coronary arteries under fluoroscopic is the most common technique to identify clinically significant plaque. However, angiography only identifies presence of plaque with no ability to assess vascular remodelling or plaque components [56]. A number of molecular probes have been developed to visualize ox-LDL in arteries [56]. IK17, the first human-derived single-chain variable fragment (scFv) antibody fragment with high specificity for oxidation-specific epitopes (OSEs) on ox-LDL, enhances imaging capability of atherosclerotic plaque. IK17 has hypoimmunogenic characteristics due to its small size, lack of fragment crystallizable (Fc) regions and as it is derived from human mononuclear mRNA antibody libraries rather than a murine source [67]. IK17 can be bonded with manganese and gadolinium in micelles to enhance the resolution of magnetic resonance imaging (MRI), resulting in high-quality imaging with specific uptake in atherosclerotic lesions in experimental animals [68]. Li et al. reported an alternative strategy with liposomes conjugated with LOX-1, an ox-LDL scavenger receptor, which are used to detect atherosclerotic lesions by single-photon emission computerized tomography (SPECT) and MRI [69].

Ox-LDL also has potential to target delivery of therapeutic nanoparticles to atherosclerotic lesions. There are few reports of targeted drug delivery to the atherosclerotic plaque, however none, to date, have directly targeted ox-LDL. Duivenvoorden et al. used HDL nanoparticles containing lipophilic simvastatin to target atherosclerotic lesions in apo E-knock-out mice. Their nanoparticles accumulated in atherosclerotic plaques, decreased lesion size on MRI, and reduced inflammation as measured *in vivo* by fluorescence molecular tomography with computed tomography (FMT-CT) and *ex vivo* by immunohistochemical analysis [70].

Despite some promise of targeting ox-LDL in preclinical studies, challenges to clinical translation remain, including identifying the most appropriate hybrid imaging targets, the safety and immunogenicity of molecular probes, and the selection of targeted drugs. There remains need for more studies.

## 4. HDL in atherosclerosis: A double-edged sword

### 4.1. HDL potentially inhibits the development of atherosclerosis

HDL is synthesized in the liver and small intestine and it formation entails production of key protein components of HDL such as apoA-I and apoA-II, acquisition of lipid and the assembly process. Although enterocytes and hepatocytes have been reported to produce apoA-I, the exact contribution of this source to overall plasma apoA-I in humans is unclear. ApoA-I synthesis by cells results in a lipid-poor form, necessitating the acquisition of free cholesterol and PLs via the ABCA1 pathway to form nascent HDL. Nascent HDL recruits more lipid from peripheral tissue and lipoproteins, which then produces cholesteryl ester produced via cholesterol acyltransferase, eventually leading to assembly of mature HDL. The liver also produces apoA-II, leading to the formation of HDL with both apoA-I and apoA-II [71].

Understanding the interactions between HDL and vascular cells has progressed over recent decades. HDL promotes endothelium and NO-dependent relaxation in wild-type but not SR-BI-knockout mice demonstrating that circulating HDL binds to the scavenger receptor BI expressed in endothelium, resulting in the simulation of eNOS [72, 73]. HDL-stimulated NO secretion may be regulated by the HDL-associated lysophospholipids sphingosylphosphorylcholine (SPC), sphingosine-1-phosphate (S1P), and lysosulfatide (LSF).

Anti-atherogenic properties of HDL particles are mainly facilitated by reverse cholesterol transport (RCT), a catabolic mechanism transferring cholesterol from peripheral tissues to the liver for excretion. Anti-inflammatory and antioxidant effects of HDL are essential for vascular protection [3]. Current clinical guidelines state that serum HDL level < 40 mg/dL are a risk for heart attack and stroke [51], whilst a healthy level for HDL is >60mg/dL, highlighting the positive role HDL plays in protection from atherosclerosis.

HDLs are highly heterogeneous resulting in various subtypes of HDL. Some sub-types of HDL demonstrate multiple antiatherogenic effects including transport of cholesterol from the arterial wall to the liver and anti-inflammatory, anti-infectious, antioxidative, anti-apoptotic, antithrombotic and vasodilatory actions [74]. However, the inherent heterogeneity of HDL results in variable results when reporting the impact of HDLs during atherosclerosis development. Johansson et al. reported that HDLs can be grouped into large (9.4 to14 nm), medium (8.2 to 9.4 nm) and small (7.3 to 8.2 nm) HDL particles but notes limitations of precision of measurement for each of these subpopulations [75]. They showed that large HDL particles (9.4 to14nm) are inversely related to coronary disease risk [75]. In contrast, other studies have shown that only the small diameter particles (7.3 to 8.2 nm) with disk-like or mature spherical HDLs can effectively remove cholesterol and enhance the antioxidant and anti-inflammatory functions whilst the larger-diameter HDLs are less effective [76, 77]. Further studies are needed to clarify the role of sub-types and different sized HDLs in the development of atherosclerosis. Despite these discrepancies, it is generally accepted that HDL particles have anti-atherogenic effects. Other beneficial effects include HDLs ability to reduce generation of endothelial adhesion molecules (Von Willebrand factor, and platelet-activating factor), and promote vasorelaxation and endothelium proliferation through stimulating endothelium to produce prostacyclin and nitric oxide (NO), thereby reducing inflammatory cell infiltration and maintaining stabilization of endothelial cells [78]. HDL can also inhibit the production of lipid hydroperoxides that play a major role in oxidizing phospho-lipids and cholesterol [79]. In addition, HDL contains several antioxidant enzymes, including paraoxonase (PON) or platelet-activating factor acetyl-hydrolase (PAF-AH). These enzymes can reduce the formation of ox-LDL, leading to the reduced deposition of ox-LDLs in vessel walls [80, 81]. Furthermore, the antioxidant activity of HDL promotes cholesterol efflux, which alleviates atherosclerosis progress [82].

Several studies have demonstrated that HDLs anti-atherogenic properties are strongly associated with its structural components, including apolipoprotein A-I (apoA-I), apolipoprotein J (apoJ), apolipoprotein E (apoE), PON, glutathione peroxidase, and PAF-AH. ApoA-I, a fundamental protein of HDL, exhibits multiple antioxidant functions. ApoA-I assists in the discharge of cholesterol from vascular macrophages and peripheral tissues via interacting with the ATP-binding cassette (ABC) A1 transporter (ABCA1) [83]. ApoA-I also collects and removes LDL lipid hydroperoxides and peroxides that could oxidize the phospholipids portion of LDLs, thus slowing down the progression of artherosclerosis by reducing ox-LDL [84-86]. ApoA-I can also bind with cholesteryl ester transfer protein (CETP) and trigger lecithin-cholesterol acetyltransferase (LCAT) to promote the RCT process to accelerate peripheral cholesterols back to the liver, thereby achieving its anti-atherosclerotic effects [87]. LCAT can also convert the nascent discoidal-shaped HDL into a mature spherical-shaped HDL particle.

Apolipoprotein A-II (apoA-II) is the second most abundant major apolipoprotein of HDL, but its specific function remains unclear. ApoA-II knock-in rabbits (without apoA-I) showed resistance to the development of atherosclerosis, when compared to wild-type rabbits (that have apoA-I only) demonstrating that apoA-II may inhibit progression of atherosclerosis. This may be related to apoA-II ability to increase plasma HDL-C and reduce triglycerides and atherogenic lipoproteins levels [88]. Results of this study suggests targeting apoA-II may be a new strategy for treatment of atherosclerosis.

ApoE is another key component of HDL exhibiting vascular protection. It has been reported as the most abundant protein in HDL isolated from atherosclerotic lesions [89], indicating an important role of apoE in atherosclerosis pathophysiology. Song et al. reported that, compared to normal controls, apoE knockout mice produce less HDL in the liver with significantly reduced expression and translation of RCT-related genes (ApoA-I and ABCA1), which may lead to the insufficient clearance of peripheral cholesterol, resulting in accelerated development of atherosclerosis [90]. Similarly, Kypreos et al. reported that apoE promotes HDL biogenesis by interacting with ABCA1, independently of apoA-I in mice [91]. When compared to HDL without apoE, apoE enriched HDL is active in size expansion and contraction and is cleared rapidly from the circulation [92]. HDL size expansion likely reflects cholesterol absorption, the primary lipid constituent of the HDL core. Conversely, HDL size contraction mainly results from selective cholesterol ester liver uptake through interacting with lipoprotein lipase (LPL) and hepatic lipase (HL) to generate prebeta-1 HDL as well as increased clearance rates from the circulation [92]. These pathways and key metabolic steps in RCT to protect against atherosclerosis, are not observed for HDL without apoE.

ApoJ, also known as clusterin (CLU), is a multifunctional protein. It is involved in a variety of physiological processes relevant to lipid transportation and vascular SMC differentiation, immune system regulation, oxidative stress, cell adhesion, apoptotic cell death, cell-cycle regulation and tissue remodelling [93]. Importantly, apoJ prevents the development of atherosclerosis. Like apoA-I and apoE, apoJ promotes RCT from peripheral tissues to the liver and accelerates phospholipid and cholesterol export from macrophage-foam cells, the hallmark cell type of atherosclerotic lesions [94, 95].

HDL surfaces have enzymes such as PAF-AH, LCAT [96], PON, and glutathione phospholipid peroxidase (GPX) [82], which all play significant roles in antioxidation. PAF-AH is a member of the lipoprotein-relevant phospholipase A2 family, and its activity is essential to the hydrolysis of bioactive lipids (e.g., PAF oxidized phospholipids and PAF) that are involved in the pathogenesis of atherosclerosis [97]. HDL-associated PAF-AH decreases the production of ox-LDL by decreasing LDL oxidation, thus slowing down the development of atherosclerosis [82]. Vasilis et al. found that HDL-associated PAF-AH activity in individuals with hypercholesterolemia was significantly increased compared with normolipidemic individuals. In normolipidemic individuals, PAF-AH is mainly related to apoB-containing lipoproteins (e.g., as VLDL, LDL, and IDL), with <20% present in HDL, but in hypercholesterolemia PAF-AH activity distribution between LDL and HDL is altered [98]. Gradient ultracentrifugation studies revealed that increased PAF-AH activity in hypercholesterolemia is because of the increasing level of plasma LDL and associated with each LDL subfraction, notably, small density LDL-5. LCAT also facilitates the metabolism of oxidatively modified phospholipids, especially those oxidatively modified phospholipids that generate during LDL oxidation, resulting in an enhanced anti-atherosclerotic effect. LCAT also plays a major role in maintaining normal HDL levels and structures. It catalyzes the transformation of prebeta HDL into mature alpha HDL, representing the majority of HDL components in the plasma [99, 100]. PON, a calcium-dependent enzyme of relevance to HDL [101], can also inhibit LDL oxidation and LDL-induced monocyte chemotactic activity to protect HDL against oxidation, resulting in the antiatherosclerotic effect [102]. Studies show that individuals with low PON activity are exposed to elevated oxidative stress [103], leading to the excess production of ROS that aggravates the atherosclerotic endothelial injury, ox-LDL formation, and lipid deposition, which promotes the atherosclerosis development process. PON can also stimulate the efflux of HDL-mediated macrophage cholesterol using the ABC A1 transporter to mediate atherosclerotic lesions [104]. Lastly, GPX inhibits hydroperoxides of phospholipids and cholesteryl esters associated with intact lipoproteins and efficiently eradicates free radicals in the body, protecting endothelium against oxidative damage and reducing lipid infiltration, thereby alleviating atherosclerosis pathological changes to some extent [105].

### 4.2. Dysfunctional HDL facilitates the development of atherosclerosis

It is traditionaly believed that the increasing levels of HDL could reduce the risk of developing atherosclerosis. New evidence suggests that the level of HDL and the risk of the developing atherosclerosis has a positive correlation, and that raising HDL levels can increase the risk of developing atherosclerosis. The 2006 ILLUMINATE clinical trial used Torcetrapib, a novel selective cholesteryl ester transfer protein (CETP) inhibitor, to improve HDL plasma levels in patients with ASCVD, and showed that the all-cause mortality increased with elevated HDL levels [106]. Navab et al. showed that elevated plasma HDL levels might contribute to aggressive development of atherosclerosis in animal models [80]. Other studies support the hypothesis that HDL may not always be anti-atherosclerotic [107, 108]. Thus, specific mechanisms of HDL require further study. Contradictary results noted above may relate to the newly discovered dysfunctional HDL that results from the alteration of structure and function secondary to oxidative modifications. In addition, heterogeneity of the HDL structure and components, including presence or not of proteins such as apoC-III, likely contributes to its variable function and effects on development of atherosclerosis.

#### 4.2.1 Oxidative modification of HDL

A number of pathways produce dysfunctional HDL including copper oxidation of HDL. Copper-oxidized HDL stimulates ROS pathways to enhance inflammation, promote apoptosis of endothelial cells, and induce the oxidation of apoproteins [82, 109-111], all of which can increase the risk of developing atherosclerosis. Oxidative modifications of HDL occur via a variety of other enzymatic pathways. Several members of the matrix metalloproteinase (MMP) family can cleave apoA-I of HDL to disrupt cholesterol efflux from foam cells [112]. Myeloperoxidase (MPO) produced by activated phagocytes is another common enzyme found in atherosclerotic lesions [113]. MPO acts on HDL, reducing the anti-atherosclerosis properties of HDL. In contrast to copper oxidation of HDL, MPO likely oxidises HDL via hypochlorous acid (HOCl), but the exact mechanism requires clarification [114]. MPO-modified HDL inhibits cholesterol-efflux by disrupting HDL binding to the scavenger Receptor-BI (SR-BI) [115]. MPO also generates tyrosyl radicals that lead to lipid peroxidation and lipoprotein cross-linking [87], thereby reducing the RCT capacity of normal HDLs and promoting cholesterol deposition in the sub-intimal space [116]. MPO can also bind to the endothelium and directly consume NO produced by endothelial cells, impacting NO signalling and results in impaired endothelial function [87].

Other enzymes that alter HDL and disrupt cholesterol efflux include tryptase, chymase, endothelial lipase, and polymorphonuclear (PMN)-derived enzymes. Tryptase and chymase are both derived from mast cells and have similar mechanisms of action. Tryptase can hydrolyze apoA-I in HDL particles, thus impairing apoA-I-mediated cholesterol efflux from foam cells [87, 117]. Similarly, chymase degrades the apolipoproteins of HDL, resulting in the oxidation of HDL, which leads to the reduction of cholesterol efflux through ABC A1 transporter [118]. Endothelial lipase impairs cholesterol efflux by elevating non-esterified fatty acids and lysophosphatidylcholine [119]. Meanwhile, the elastase derived from PMN damages cholesterol-efflux by producing ROS species [120]. Acute-phase proteins, including serum amyloid A protein (SAA) and sPLA2, can also play a major role in the formation of dysfunctional HDL. SAA binds to HDL, causing impaired access of HDL to the plasma membrane, thereby decreasing cholesterol efflux [121]. SAA can also block HDL-relevant enzymes such as PON and PAF-AH, nullifying their ability to resist LDL oxidation [122]. sPLA2 hydrolyses HDL phospholipids, resulting in disrupted cholesterol efflux [123].

Patients with type 1 diabetes often have severe atherosclerosis despite the increased levels of HDL [124]. This may be related to increased glycation promoting ROS production, oxidation, and inhibition of NO system, causing the HDL structural alterations and functional disorders. Ferretti et al. examined the effect of incubating HDL under hyperglycaemic conditions on lipid composition and PON activity and showed that modification of the polarity of glycated HDL affects HDL-associated PON activity, which may contribute to the accelerated atherosclerosis progression in diabetic patients [125]. Hedrick et al. incubated human HDL (5 mg protein) with or without 25 mmol/l glucose *in vitro* and found that *in vitro* glycated HDL did not inhibit monocyte adhesion induced by ox-LDL to human endothelium and caused a 40% reduction in PON activity. They also found a 40% reduction in PON activity in patients with Type II (non-insulin-dependent) diabetes mellitus and atherosclerosis compared with non-diabetic subjects. Based on these results, they propose that dysfunctional glycated HDL could contribute to the accelerated development of atherosclerosis in Type II diabetes patients [126].

Taken together, multiple pathways can form dysfunctional HDL, but studies are limited to *in vitro* and animal studies. Thus, the significance of HDL modifications in the human health and disease remains unclear.

#### 4.2.2 Certain intrinsic components of HDL may possess pro-atherogenic properties

Despite the generally accepted role of HDL in inhibiting or slowing atherosclerosis, some components of HDL may accelerate atherosclerosis. Apolipoprotein C-III (apoC-III) is an example as Allyson et al. (2018) have shown that, compared with HDL lacking apoC-III, HDL with apoC-III is associated with a higher risk of CVD. HDL containing apoE generally protects against cardiovascular risk, but apoC-III in HDL negates the beneficial effect of apoE on HDL metabolism and relation to cardiovascular disease [92].

## 5. Cutting-edge therapeutic approaches that target lipoprotein metabolism

**Figure 2.**
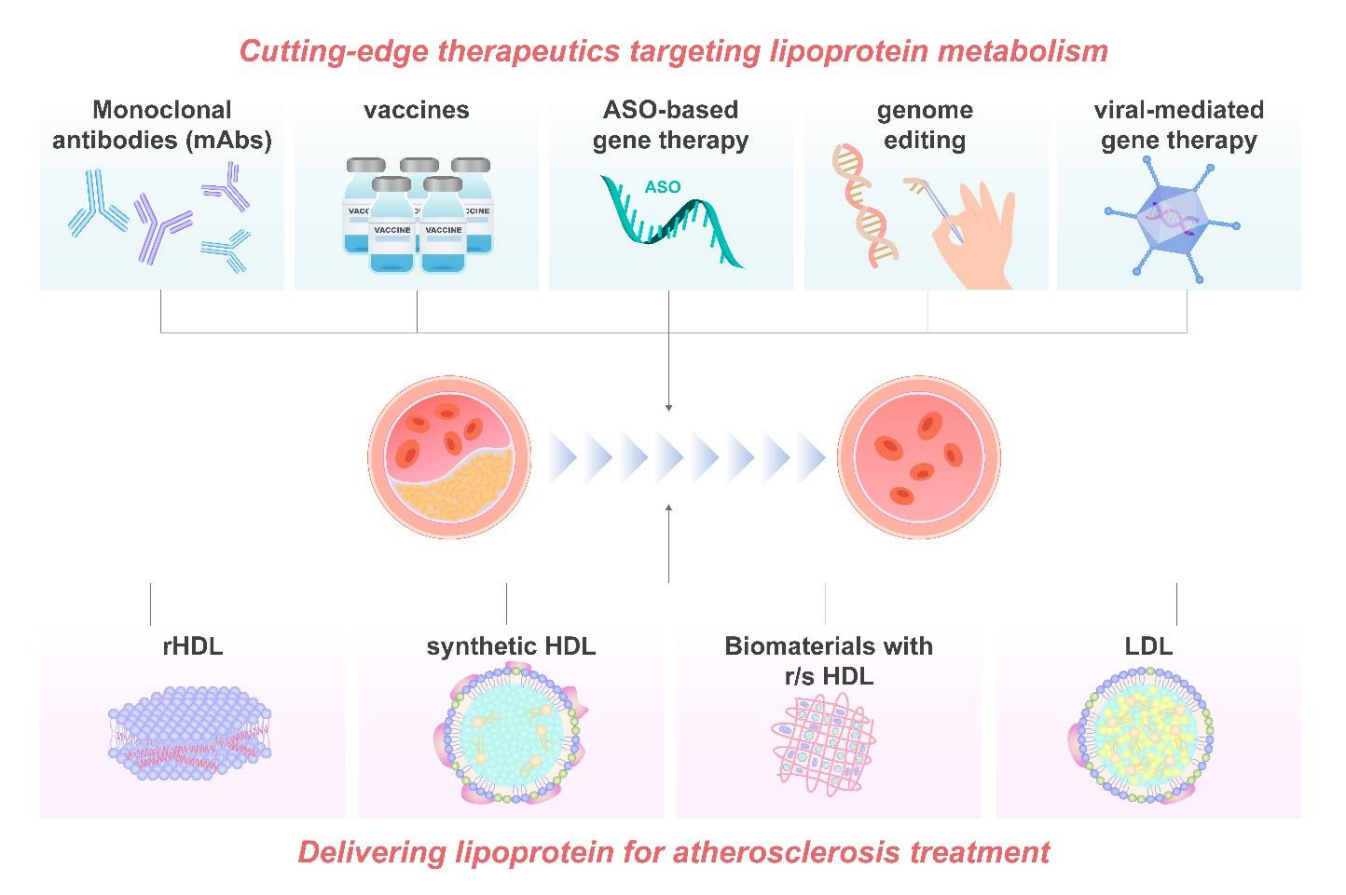
Summary of targeting and delivering therapies on atherosclerosis treatment. To resolve the limitations in the efficacy and safety of existing treatment methods of atherosclerosis, biological drugs with improved targeting are currently being explored. One of the most promising directions is to target lipoprotein metabolism. Monoclonal antibodies (mAbs), vaccines, antisense oligonucleotide (ASO)-based gene therapy, genome/base editing technologies and viral-mediated gene therapy are all cutting-edge therapeutic approaches that target lipoprotein metabolism. Another advanced therapy strategy is to delivery natural or synthetic lipoproteins for the treatment of atherosclerosis. Reconstituted (r) HDL has been widely accepted as an ideal drug delivery vehicle, because of their nano-size, unique cellular uptake mechanism via a non-endocytic pathway. Synthetic HDL (sHDL), using a nanoparticle template to tailor the structure and the chemical composition of the HDLs, is featured with improved size, shape and surface chemistry and with less batch-to-batch variation. Biomaterials combined with r/s HDL may have enhanced therapeutic efficacy. LDL could be applied as a vesicle to delivery targeted therapeutic drugs.

In order to improve efficacy and safety and to achieve more precise and targeted delivery of existing treatments, interest is growing in new strategies targetting lipoprotein metabolism. A number of lipid-lowering therapies have FDA approval and are in routine clinical practice with other promising agents in pre-clinical or clinical trials [127]. Figure 2 and Table 1 summarize cutting-edge therapies and delivery strategies targeting lipoprotein metabolism to treat atherosclerosis.

**Table 1:** Cutting-edge therapeutic approaches that target lipoprotein metabolism.

Category	Strategy	Target	Method	Models	Therapeutic effect	Refs.
mAbs		PCKS9	PCKS9 silencing	*In vitro* and apoliporotein E knockout mice	(1) proinflammatory cytokine downregulation (2) TLR4/NF-ĸβ inhibition	[129]
			Alirocumab	APOE*3Leiden.CETP mice.	(1) decreasing the lipid component of non-culprit plaques (2) stabilize plaque	[130, 132]
			Alirocumab	Clinical trial	(1) reduced mortality (2) reduced risk of stroke and myocardial infarction (MI)	[131]
			Bocociziumab	Parallel and randomized clinical trials	(1) reduced the LDL levels in most patients with hyperlipidemia at 3 months	[133]
			Evolocumab	Clinical trials	(1) reduce the cholesterol levels by an average of 0.7mmol/l (2) reduce the risk of cardiovascular events	[135]
		ANGPTL3	Evinacumab	Phase 2 clinical trial	(1) 49% reduction in LDL-C (2) <80% reduction in triglycerides	[143]
			Evinacumab	Clinical trial	(1) benefit for patients with LDL receptor mutations (2) 34% reduction in LDL-C of patients with biallelic null LDL receptor mutations	[144]
Vaccine	Cholesterol lowering	PCKS9	Inclisiran	Phase III clinical trial	(1) inhibition of PCKS9 (2) 50% reduction in LDL-C (3) injection-site adverse events	[155, 156]
			Peptide based AT04A	APOE*3Leiden.CETP mice	(1) reduction in plasma lipids (2) decreased inflammatory response (3) diminished atherosclerotic lesions	[157]
		ApoC3	ISIS 304801 (Antisense DNA)	Clinical trial	(1) decrease in triglyceride levels	[158]
			VLPs	Mice	(1) reduction in plasma lipid level	[159]
		CETP		Rabbit	(1) 24% reduction in LDL-C (2) 39.6% reduction of atherosclerotic lesions	[160]
			CETi-1	Phase I clinical trial	(1) insignificant reduction in HDL	[161]
	Antigen-inducing	HSP65	Lactococcus lactis	LDL receptor deficient mice	(1) upregulation of IL10 (2) downregulation of IFN-γ (3) atheroprotection	[163]
		HSP60	Porphyromonas gingivalis	Hyperlipidaemia (Apoeshl) mice	(1) increase in IL-10 (2) reduction in CRP, MCP-1. Ox-LDL (3) inhibition in atherosclerotic lesion formation	[164]
		Ox-LDL		Hypercholesterolemia rabbits	(1) reduction in atherosclerotic lesion size (2) increase in ox-LDL antibodies	[152]
		LDL ox-LDL		Hypercholesterolemia rabbits	(1) ox-LDL antibodies increase in both LDL and ox-LDL immunized rabbits (2) 74% reduction in atherosclerotic lesions in LDL immunized rabbit (3) 48% reduction in lesions in ox-LDL immunized rabbit	[153]
		ox-LDL LDL		Hypercholesterolemia rabbits	(1) 58% reduction in the neointial area in ox-LDL immunized rabbit (2) 19% reduction in the neointimal area in LDL immunized rabbit (3) reduced T cells and ox-LDL in ox-LDL immunized rabbit	[165]
		MDA-LDL		Apo-E-deficient mice	(1) upregulation of MDA-LDL antibodies (2) reduced lesion size at the aortic sinus	[166]
		ApoB-100	P210	ApoE-Null Mice	(1) 60% reduction in atherosclerotic lesion (2) increase in collagen content of subvalvular lesions	[168]
			P45, P74	ApoE deficient mice	(1) P45 reduced the atherosclerosis by 48% and reduced the macrophage in lesion by 33% (2) P74 decrease the lesion by 3% and macrophage content by 39%	[170]
	Multitarget	Apob-100 HSP60	P45 and Chylamydophia pneumonia	Ldlr-/-mice	(1) reducing lesion size wihouth Cpn infection (2) downregulation of cellular infiltration, and inflammatory cytokine/chemokine section	[172]
Gene-therapy	ASO based	ApoB mRNA	Mipomersen	Clinical trials	(1) 24.7% reduction in LDL-C (2) adverse effects: flu like side effect, liver steatosis, injection site reaction	[173]
		Apo(a) mRNA	IONIS-APO(a)-LRX	Clinical trials	(1) 71.6% reduction in Lp(a) level	[178]
			AKCEA-APO(a)-LRx (TQJ230)	Clinical trial Phase III	80% reduction in Lp(a) level	[179]
		ApoC3 mRNA	Volanesorsen	Phase III clinical trial	77% decrease in triglycerides	[180]
		ANGPTL3	IONIS-ANGPTL3-LRX	Phase II clinical trial	(1) 33.2-63.1% reduction in triglycerides (2) 1.3-32.9% reduction in LDL-C (3) 27.9-60% reduction in VLDL-C	[181]
	Viral mediated	LPL	Glybera (AAV1)	Clinical trial	(1) reduced pancreatitis events (2) decreased acute abdominal pain events associated with panceratitis (3) discontinued	[185]
		LDLR	AAV8	Humanized mouse models of familial hypercholesterolemia	Significant reduction in LDL-C level	[188]
				Humanized mouse model of familial hypercholesterolemia	(1) reduction in plasma cholesterol and non-HD-C level (2) 87% reduction in lesions after 3 months (3) significant remodelling of lesion	[187]
				Phase I clinical trial	(1) limited toxicity and no signification upregulation of pro-inflammatory cytokines	[189]
		ApoA-I	HD-Ad	Apo E-deficient mice	(1) upregulation of APOA-I expression and HDL-C (2) reduction in lesion size	[191]
				Rabbit	(1) 70% reduction in plasm cholesterol (2) 208% reduction in VACM-1 expression (3) 30% reduction in macrophage content (4) reduction in lesion size, lipid content and ICAM-1 expression	[192]
				Hyperlipidemic rabbits	(1) apoA-I mRNA expression (2) reduction in lesion size, lipid/macrophage content (3) downregulation of adhesion molecule expression	[193]
				Hyperlipidemic rabbits	(1) 30% reduction in intimal lesion volume (2) 23-32% reduction in intimal lipid, macrophage and SMCs contents (3) 36% reduction in VACM-1 expression (4) downregulation of ICAM-1, MCP-1 and TNF-α	[194]
	Genome/base editing	LD-R	AAV-8 mediated CRISPER/Cas 9	Adult mice	(1) development of hypercholesterolemia adds atherosclerosis	[201, 202]
		PCKS9	S. pyogenes Cas9 in adenoviral vector	Mice	(1) decrease in PCSK9 level (2) increase in hepatic LDL receptor level (3) 35-40% reduction in serum cholesterol levels	[203]
			aureus Cas9 in AAV	Mice	(1) over 40% gene modification (2) reduction in PCSK9 and cholesterol levels	[204, 205]
			Streptococcus pyogenes Cas9	Mice	reduced peptides, decreased indels and no chromosomal translocation in addition to reduced TC levels	[203]
		ANGPTL3		Mice	(1) 56% reduction in triglycerides (2) a 51% decrease in LDL-C	[207]

### 5.1. Monoclonal antibodies (mAbs)

Monoclonal antibodies (mAbs), targeting PCSK9, angiopoietin-like protein 3(ANGPTL3), angiopoietin-like protein 4(ANGPTL4), ApoC3, and ApoB-100 peptides, are the dominant lipid-lowering medications in current pre-clinical and clinical research.

The most popular of these targets, PCSK9, is produced by the liver and targets the LDL-receptor (LDLR) on the surface of hepatocytes, resulting in the inhibition of LDL clearance and increased LDL levels. When PCSK9 is inhibited, LDLs recycle to the cell surface, with improved clearance from circulation. Therefore, loss-of-function mutations of PCSK9 have relevance to decreased LDL levels and reduced CHD risk [128]. PCSK9 inhibitors also regulate inflammatory responses by downregulating the expression of pro-inflammatory cytokines. In particular, the TLR4/NF-ĸB pathway is associated with PCSK9 induced pro-inflammatory cytokines such as IL-6, IL-1, TNF-α, IFN-γ and MCP-1 [129]. PCSK9 mABs have also been demonstrated to stabilize plaque via decreasing the lipid component of non-culprit plaques [130-132]. Current clinically available PCSK9 inhibitors include bococizumab, evolocumab, alirocumab, and LY3015014 (LY) [131], which all mediate LDL levels and prevent or slow the development of ASCHD and other CVDs. Bocociziumab has been shown in six parallel randomized control trials to significantly reduce LDL levels in most patients with hyperlipidemia at 3 months [133]. Both bococizumab and alirocumab showed improved reduction of Lp(a) compared with statins [121, 134]. Evolocumab reduced cholesterol levels by an average of 0.7mmol/l, significantly reducing cardiovascular event rates with median follow-up of 2.2 years [135]. Several clinical trials of evolocumab or alirocumab administered to patients with recent acute coronary syndrome (ACS) or stable ASCVD and receiving maximum tolerated statin therapy showed reduced risk of stroke and myocardial infarction (MI) [136, 137]. Taken together, PCSK9 inhibitors are recommended as additional or alternative therapy in patients with complete statin intolerance ASCVD and familial hypercholesterolemia (FH) with persistent hypercholesterolemia [138-140].

ANGPTL3 was demonstrated as playing a key role in lipid metabolism by studies in a group of strain KK obese mice, where ANGPTL3 was associated with hyperinsulinemia and hyperglycemia [141]. ANGPTL3 gene plays an important role in modulating TC, LDL, HDL levels by inhibiting lipoprotein lipase (LPL) and endothelial lipase (EL) activities via its coiled-coil region [142]. In phase 2 clinical trials, evinacumab, an ANGPTL3 inhibitor, reduced LDL-C by 49% and decreased triglycerides up to 80% [143]. Evinacumab is particularly beneficial for patients with LDL receptor mutations, as its effects to reduce LDL-C are independent of LDL receptors as demonstrated by a 34% reduction in LDL-C of patients with biallelic null LDL receptor mutations [144]. Thus, evinacumab is suitable for patients with severe heterozygous familial hypercholesterolemia.

ANGPTL4, an endogenous inhibitor of LPL, can modulate LDL, HDL, and reduce the risk of coronary atherosclerosis. Interestingly, ANGPTL4 glycoprotein shares 31% of the same amino acid sequence with ANGPTL3, and both genes have an identical modular structure [145]. However, the transcriptional regulation of the two genes is different. ANGPTL3 is regulated by LXRs and HNF1α [146]; meanwhile, ANGPTL4 is regulated by feeding and fasting [147]. That being said, both genes inhibit LPL activity and increase TG levels. However, the specific role of ANGPTL4 in atherosclerosis progression is controversial. Aryal et al. reported that the inhibition of ANGPTL4 in haematopoetic cells can improve monocyte expansion and promote foam cell formation via CD36 upregulation and ABCA1 localization reduction, resulting in the progression of atherosclerosis [148]. Similarly, ANGPTL4 reduces the inflammatory responses and decreases the number of immune cells (monocytes and macrophages) accumulating in the atherosclerotic plaque in ANGPTL4 TG E3L mice, indicating the upregulation of ANGPTL4 expression can prevent or slow the progression of atherosclerosis [149]. In contrast, Adachi et al. found that the inhibition of ANGPTL4 enhanced lipid metabolism and reduced foam cell formation, resulting in the protection against atherosclerosis in ApoE^-/-^ ANGPTL4^-/-^ mice [150]. Nevertheless, evidence indicates that ANGPTL4 does play a vital in atherosclerosis development, but the mechanism requires clarification. There are no clinical drugs targeting ANGPTL4 to date, but it may offer potential as a target in future.

Taken together, mAbs based drugs offer clinical utility due to their ease of administration, therapeutic efficacy and synergy in working with statins. However, certain limitations of mAbs therapies impede further downstream commercialisation. As an example, the PCSK9i bococizumab was suspended in 2016 due to unsatisfactory safety and efficacy in phase III trials. In addition, mAb therapies such as PCSK9i have issues with affordability and availability.

### 5.2. Vaccine

Atherosclerosis can be considered a chronic inflammatory condition due to the involvement of monocytes, macrophages and T lymphocytes in atherosclerotic plaque formation. Vaccines against specific antigens effectively target the immune response in a number of autoimmune diseases including atherosclerosis [151]. Palinski et al. (1995) first demonstrated the administration of malondialdehyde (MDA)-modified lysine effectively generated high titers of antibodies resulting in reduced atherosclerotic burden [152]. Ameli et al. showed antibodies against LDL and ox-LDL decreased atherosclerotic lesions by 74% and 48%, respectively in a rabbit model [153]. Since those early reports, increasing numbers of studies have shown vaccination against a number of targets, including cholesterol, active immunization against plaque antigens and multitarget strategies, can effectively treat atherosclerosis [154].

As seen with mAb therapy, a key target for immunization is PCSK9. Inclisiran, a siRNA-based drug to inhibit the expression of PCSK9, has shown efficacy in phase III trials that enrolled over 3000 patients with ASCVD and 482 patients with familial hypercholesterolemia [155, 156]. Other vaccines, including a peptide-based vaccine and AT04A, targeting PCSK9 have shown promise in preclinical animal models. Another target for lowering cholesterol is apolipoprotein CIII (apoC3) [157]. Antisense DNA that targets apoC3 successfully decreased triglyceride levels (TG) in patients with hypertriglyceridemia [158]. A virus like particle-based vaccination strategy targeting apoC3 has shown promise in reducing TG levels in mice [159]. Vaccine-induced CETP inhibition has been shown to reduce aortic lesions in a rabbit model [160]. However, phase I clinical trials of the CETP vaccine did not lower HDL levels as expected [161].

As plaque-associated antigen-induced immunogenic responses play a vital role in atherogenesis, autoantigens could be targeted to prevent atherosclerosis. Heat-Shock proteins (HSPs) are a popular target due to their role in inhibiting the denaturation or loss of function of proteins when cells are under stress. HSP60 expression increases in endothelial cells when atherosclerotic risk factors are present [162]. Oral immunization against HSP65 was developed to downregulate expression of HSP65 leading to upregulation of IL-10 [163]. A vaccine against HSP 60 was atheroprotective in spontaneously hyperlipidemic (Apoe^shl^) mice [164]. Further HSP based immunization targets are being developed with LDL, oxLDL and apoB based vaccines being tested preclinically. A vaccine against oxLDL administered to hypercholesterolemic rabbits reduced atherosclerotic lesions associated with upregulation of ox-LDL antibodies [152]. LDL based vaccines also achieved atheroprotection in rabbits and mice [153, 165, 166]. Identifying immunogenic epitopes is essential to effective vaccination. Comparison of apoB based peptides to human apoB-100 identified 102 different apoB like peptides that could be a target for immunization [167]. Numerous other studies have targeted immunization against peptides such as native p143, p210 [168], p2 [169] and MD-modified p45 and p47 [170].

Multitarget strategies use more than one vaccine simultaneously, either targeting a single pathogen through multiple serotypes or to target multiple pathogens [171]. As an example, Lu et al. used a mixture of apoB (p45), HSP60, and *Chylamydophia pneumonia* as a multitarget vaccine in Ldlr^-/-^mice. This promoted a significant Treg response resulting in improved atheroprotection compared to a single vaccine [172].

Overall, vaccines raised to atherogenic-related antigens exhibit unique advantages, but further studies are required to focus on durability of immunization, efficacy, safety and side effects.

### 5.3 Gene-based therapy

The recent success in mRNA-based vaccine strategies for COVID-19 shines a light on the potential of gene-based therapies. Through downregulation or upregulation of specific gene expression, such therapies may present a new paradigm for treatment of atherosclerosis.

#### 5.3.1 Antisense oligonucleotide (ASO)-based gene therapy

Synthetic single-stranded DNA or RNA oligonucleotides combined with a specific complementary mRNA species have been applied to promote degradation of the specific complementary mRNA (ribonuclease H1-dependent class) or to disrupt its translation or splicing (steric-blocker class). This has been trialled for ASOs targeting apoB, apoC3, ANGPTL3, Lp(a), and PCSK9.

Mipomersen, the first FDA (2013) cardiovascular ASO drug to be approved by, targets the coding region of the apoB mRNA and reduces LDL levels in patient’s intolerant of statin therapy and at high risk of CVD, and in homozygous/heterozygous FH patients with pre-existing CHD [173-175]. In a Phase III clinical trial, 26-week treatment with mipomersen demonstrated a 24.7% decrease in LDL-C level (NCT00607373). However, severe adverse effects including flu-like symptoms, increased risk of liver steatosis and injection site reaction resulted in mipomersen being withdrawn from the market in 2019 [176-177].

Another ASO based drug, IONIS-APO(a)-L_RX,_ targets Apo(a) mRNA resulting in cleavage and inhibition of Lp(a). Given the absence of other clinically approved therapies that lower Lp(a), this new drug shows promise in reducing Lp(a) levels and preventing the development of atherosclerosis. Weekly injection of 300mg IONIS-APO(a)-L_RX_, reduced Lp(a) levels by 71.6% [178]. A second-generation ASO targeting Lp(a), AKCEA-APO(a)-LRx, also named TQJ230, demonstrates high efficacy and specificity to reduce Lp(a) levels [179]. Weekly administration (20 mg) reduced Lp(a) levels 80% with improved therapeutic effect. This drug is now in Phase III clinical trial.

Volanesorsen targets mRNA for apoC, the lipoprotein that inhibits LPL, removes hepatic TCL, and contributes to hypertriglyceridemia. In a Phase 3 trial, weekly injections of 300 mg Volanesoren reduced triglycerides by 77% without notable side effects [180].

IONIS-ANGPTL3-L_RX_, an ASO targeting the ANGPTL3 gene, in Phase I clinical trial showed significant reduction in triglycerides (33.2-63.1%), LCL-C (1.3-32.9%) and VLDL-C levels (27.9-60%) [181]. The drug has progressed to Phase II trial.

A significant challenge for ASOs is optimizing precise targeted delivery. Prakash et al. proposed conjugating ASOs to N-acetyl galactosamine (GalNAc), a high-affinity ligand for the hepatocyte-specific asialoglycoprotein receptor, which enhanced delivery to hepatocytes by 10-fold [182]. A large number of other conjugated moieties are also being explored [183].

#### 5.3.2 Viral-mediated gene therapy

Aside from ASOs reviewed above, viral mediated gene therapies, delivering DNA coded to interfere with transcription in order to compensate for abnormal genes and improve the expression of beneficial proteins, have also been developed for treatment of atherosclesis [184]. Adeno-associated viruses (AAVs), the most common viral vectors, are highly effective for transfection and can transduce both dividing and non-dividing somatic cells. Their simple structures and good safety profiles make them ideal vehicles for gene-based therapies.

Glybera®, the first gene therapy product targeting atheroscletosis, is an AAV1 vector carrying an intact copy of LPL aimed at reversing the severe hyper-triglyceridemia-causing LPL deficiency [185]. However, it was discontinued two years later on financial grounds [186]. A recombinant AAV8 vector containing a mouse or human LDLR transgene given to mouse models of FH resulted in stable dose-dependent reduction in non-HDL-c and TC, with decreased inflammatory cell infiltration and atherosclerotic plaque formation, and favourable plaque remodelling [187-189]. The vector is well tolerated when delivered at clinically relevant doses to mouse and rhesus macaque [189, 190] and it is now undergoing early clinical trial for efficacy and safety (NCT02651675).

Helper-dependent adenoviral vectors (HDAd) lack all viral coding sequences, and hence overcome the disadvantage of the high immunogenicity of other vectors. HDAd vector expressing apoA1, the primary protein of HDL, given to rabbits and mice results in significant reduction of atherosclerotic lesions with negative hepatotoxicity [191-194].

Despite progress in viral-mediated gene therapy, challenges remain. These include dominant mutations, unintended effects on neighbouring genes, risk of insertional mutagenesis, the size limitation of viral vectors and low efficacy of removing targeted genetic material [195]. Although the molecular targets overlap with other strategies, technical advantages and safety profile of viral-mediated methods can have benefits over other strategies for some targets [196]. There is also growing interest in the therapeutic potential of apolipoprotein mimetic peptides [197, 198].

#### 5.3.3 Genome/base editing technologies

Whereas viral-mediated gene therapy is based on a gene replacement principle, genome/base editing aims to modify DNA segments, including insertion, deletion, or replacement, in order to achieve permanent targeted DNA modifications [199]. The most extensively used tool in atherosclerosis is the CRISPR (clustered regularly interspaced short palindromic repeats)/Cas9 system to target genes such as PCSK9, ANGPTL3, apoB, and LDLR.

Genome editing is often categorised as germline or somatic gene editing. Germline editing, used in the embryonic phase resulting in the gene alteration to the offspring, has some ethical concerns as highlighted by the recent report of genetically edited girls for HIV immunization [200]. In contrast, somatic gene editing is restricted to the adult organism without transfer into the next generation.

Jarrett et al. was the first to apply this technology to atherosclerosis, using AAV8-mediated CRISPR/Cas 9 to disrupt the LD-R gene in adult mice, resulting in the development of hypercholesterolemia and atherosclerosis [201,202]. Subsequently others have investigated the potential of gene editing for treatment of atherosclerosis. Inhibition of PCKS9 in mice using *S. pyogenes* Cas9 in AAV and *aureus Cas9* in AAV resulted in a 90% reduction of PCKS9 associated with reduced cholesterol levels and limited off-target effects [203-205]. Carreras et al. showed that targeted editing of PCSK9 in the humanized mouse model induced liver-specific expression of human PCSK9 and decreased both protein and plasma total cholesterol (TC) levels resulting in a human-like hypercholesterolemia phenotype [206]. In the same model, PCSK9 base editing reduced peptides, decreased internal deletions (indels) with no chromosomal translocation and further reduced TC levels compared to genome editing, indicating that base editing may be safer and a more precise method [206]. In principle, base editing of PCSK9 avoids double-strand DNA breaks, reduces the chance of off-targeting indels and enhances on-target indels. Base editing to disrupt ANGPTL3 gene using CRISPR-Cas 9 resulted in a 56% reduction in triglycerides and a 51% decrease in LDL-C [207].

Taken together, genome/base editing technologies show potential for treatment of ASCVD. However, issues to be addressed prior to clinical translation include off-target activity, the large size of Cas, insufficient indel and homology direct repair efficiency and immune responses [208]

## 6. Advancements in strategies for delivering lipoproteins

### 6.1 HDL

#### 6.1.1 rHDL

Recombinase HDLs (rHDL) are the discoidal HDLs resulting from the bond between apoA-1 and phospholipids and consequential self-assembly. They incorporate a hydrophobic core, due to lipid bilayer formation from the hydrophilic part of the amphiphilic apoA-1 α-helices, and an aqueous tail, due to the hydrophilic portions of the amphiphilic apoA-1 α-helices. rHDL is often produced through dialysis-based synthesis where apoA-1, phospholipids and sodium cholate or other detergent are placed together, resulting in the self-assembly [209]. An alternative approach is sonication of apoA-1 with phospholipids, followed by centrifugation [210]. A recently developed microfluidic system has also been employed to synthesize rHDL in a high throughput manner, where phospholipid and a hydrophobic payload are deposited into an apoA-1 buffer, resulting in suspension formation of a phospholipid, which is then homogenized through a microfluidizer high-shear processor [211].

Direct injection of rHDL can reduce inflammation and the development of atherosclerosis in preclinical models. A recent clinical trial demonstrated a 4.2% reduction in atheroma volume 5 weeks after intravenous injection of the rHDL Apo A1Milano [212]. In patients with acute coronary syndrome, although atheroma volumes were not significantly reduced 5 weeks after infusion of rHDLs compared to placebo, there were improvements in plaque characterization and coronary scores [213]. Shaw et al. showed that rHDL injection reduces expression of inflammatory markers such as TNFα and CD11bm and reduced lipid accumulation in atherosclerotic lesions [214]. Taken together, rHDL shows therapeutic potential as a treatment for atherosclerosis.

**Figure 3.**
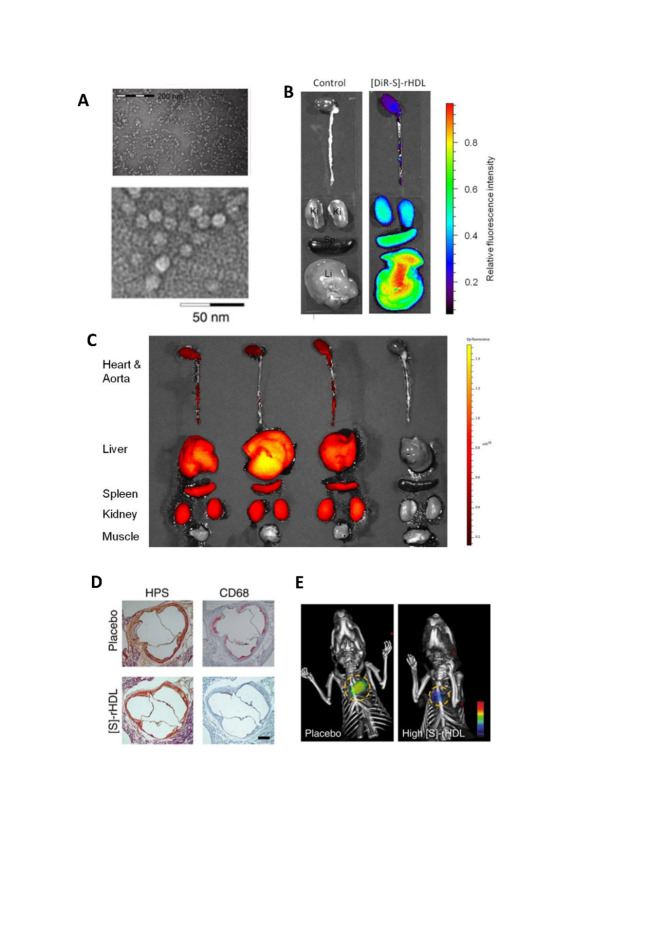
The strutural features of reconstituted high-density lipoprotein (rHDL) that promote targeted treatment. (A) Negative staining TEM image showed the typical disk-like morphology of rHDL. (B) Mice were intravenously injected with [DiR-S]-rHDL nanoparticles [rHDL fabricated with stain], and organs were imaged with NIRF 24 hours after the injection. Liver has the highest retention of DiR, followed by spleen, and kidney has the lowest retention. (C) Three mice were intravenously injected with [DiR-S]-rHDL (left three) and one control mouse was not injected (on the right). Organs were imaged with NIRF 24 hours after the injection. While heart, aorta, liver, spleen, and kidney tissue all took up nanoparticles, muscle tissue did not. (D)Typical histology images of the aortic sinus area from a mouse in the placebo group and a mouse in the high-dose [S]-rHDL group show that the mean plaque area is similar, while the plaque macro-phage content is notably smaller in the [S]-rHDL group. (E) FMT-CT molecular im-aging of protease activity revealed that high-dose [S]-rHDL treatment significantly reduced the inflammation levels in the aortic roots of live apoE-KO mice with advanced atherosclerosis as compared with placebo. The yellow circles indicate the aortic root area. All figures were cited from reference [70].

rHDL is widely accepted as a drug delivery vehicle due to its nano-size and unique cellular uptake mechanism via a non-endocytic pathway which results in improved therapeutic effect [215,216]. Furthermore, the hydrophobic core of lipoproteins can be loaded with hydrophobic drugs which are estimated to represent 40% of new therapeutic agents [217]. Simuvastatin loaded rHDL ([S]-rHDL) is a novel therapeutic for atherosclerosis [70] (Fig. 3). Statins have commonly been used to reduce LDL-cholesterol levels, but their effect is often limited to the liver. By incorporating HDL nanoparticles, the statin can penetrate the atherosclerotic plaque even when injected intravenously. Within a week of high dose [S]-rHDL, significant reductions in plaque area are observed, along with reduced number of positive macrophages. Rather than inhibiting monocyte recruitment, [S]-rHDL inhibits macrophages proliferation, together with suppression of inflammation within local macrophages. Combination treatment with [S]-rHDL injection and oral statin shows added benefit over either treatment alone [218].

Challenges remain for this treatment strategy, including attaining sufficient Apo AL donor levels, the labor-intensive isolation process and some degree of insufficiency of the intravenous infusion delivery approach [219].

#### 6.1.2 Biomaterials and synthetic HDLs

Advances are being made by using new biomaterials in the fabrication process for rHDL to improve efficacy. Hyaluronic acid (HA) has been used to encapsulate rHDL in an attempt to reduce undesirable accumulation within the liver and improve delivery to the plaque [220]. HA can reduce binding between rHDL and the SR-BI in the liver, and promote precise targeting of CD44 in plaques. HA-LT (lovastatin)-rHDL accumulates in the plaque at twice the rate compared to LT-rHDL not encapsulated with HA. Furthermore, the HA-LT-rHDL treatment group exhibited minimal macrophage activation and matrix metalloproteinase expression along with reduced atherosclerotic lesion size.

Synthetic HDL (sHDL), also known as a spherical HDL biomimetic, uses a nanoparticle template to tailor the structure and the chemical composition of HDLs, resulting in an improved consistency in size, shape and surface chemistry with less batch-to-batch variation [219]. Thaxton et al. developed sHDLs using gold nanoparticles as the core (HDL-AuNPs), similar to rHDL [221]. HDL-AuNPs bind well to cholesterol (Kd=3.8nM), which is comparable to cholesterol acceptors such as serum-derived apoA-1 and rHDL. Zhang et al. synthesized an sHDL named florescent nanocarriers (FCNs) [222]. FCNs contain DMPC, cholesterol oleate, drug, fluorescent dye and DiR-BOA. Similar to rHDL, the hydrophobic core of FCNs effectively load Dir-BOA. FCNs can bypass endosomal sequestration, due to the SR-B1 uptake. Epidermal growth factor receptor (EGFR) has also been conjugated to FCNs, allowing the EGFR-mediated endocytosis with an extended half-life (13.6 h) and targeted delivery [223].

PLGA is another material used as a nanoparticle template for sHDL synthesis. An advanced sHDL consisting of a PLGA core, a lipid bilayer structure, and HA (PLGA-HDL-HA NPs) have been developed to encapsulate simvastatin resulting in a slow-release profile [224]. Incorporating HA reduced binding to the liver, and the lipid bilayer facilitated removal of cholesterol from macrophages. In the atherosclerotic New Zealand rabbit model, sHDLs accumulated within plaque via the CD44 pathway, rather than via the liver, and were taken up by macrophages via SR-BI mediated endocytosis, resulting in improved efficacy to prevent the development of atherosclerosis. Similarly, atorvastatin (AT), has been loaded into PLAG-HDL-HA NPs with dextran sulphate to increase delivery efficiency. This enhanced cholesterol efflux and inhibited the ox-LDL take up of macrophages [225]. Further development of PLGA-HDL-HA NPs drug by incorporating apoA-1 and HA-DOPE can target macrophage and ECs sequentially. AT and lectin-like ox LDL receptor-1 small interfering ribonucleic acid were loaded into the NPs, increasing therapeutic effect [216].

Another dual-targeting strategy was developed to improve the anti-atherosclerotic effects. An adenosine triphosphate (ATP)-responsive ternary core was used, and scavenger receptor A (SR-A) was loaded to promote ATP production resulting in the accelerated release of SA-A siRNA. Dual targeting was achieved by incorporating apoA-1 and phosphatidylserine to target SR-BI and CD36 receptors. *In vivo* studies demonstrated a 3.3-fold increase in accumulation of NPs in the plaque, resulting in 65.8% plaque reduction [227].

ApoA-1 mimetic peptides have gained popularity recenty years to fabricate sHDL NPs in order reduce cost and production time compared to using apoA-I extract. sHDL NPs using synthetic apoA-1 mimetic peptides showed improved cholesterol efflux [228] and diminished the atherosclerotic lesion by 50% through oral administration in Ldlr-/- mouse model [229].

### 6.2. LDL

#### 6.2.1 LDL

Due to the high prevalence of the LDLr on malignant cells and macrophages, LDL is another potential delivery vehicle [230]. LDL has several advantages including a long half-life in the circulation, high loading capacity, improved cell uptake efficiency and penetration, and the potential for multiple loading strategies [231].

As with HDLs, the hydrophobic core of LDL facilitates drug loading. Dexamethasone has been incorporated into LDL resulting in improved delivery efficiency to plaques and slows the development of atherosclerosis in a mouse model [232, 233]. In addition to direct loading into the hydrophobic core, conjugation is also used to load drugs into LDL. Modified LDL with covalent bonds to apolipoprotein and pre-loaded with amino acid groups, including tyrosine, lysine, arginine and cysterine, has been used for *in vivo* imaging [234-236]. Modifying apolipoprotein leads to inactivation of ApoB-100 [237]. The insertion strategy utilizes the non-covalent bonding between pre-load and phospholipid layer through weak interaction such as van der Waals forces [215]. The pre-loaded drug often requires an amphiphilic structure to improve success of insertion [238].

Given its role in foam cell transformation and atherosclerotic development, ox-LDL is an ideal delivery vehicle for targeted delivery. Ox-LDL loading with dexamethasone palmitate effectively inhibits foam cell formation and significantly reduces accumulation of cholesteryl esters [239]. In contrast, LDL based carriers may aggravate disease progression due to the presence of apoB (pro-atherogenic molecules). The synthetic process of LDL is challenging and complex due to the large size of apoB, significantly limiting its potential [240]. LDL based delivery systems should be further investigated [231].

#### 6.2.2 LDL mimicking particle

LDL mimic particles (LMP) should be considered as an alternative carrier. Compared to native LDL based carriers, LMP can be synthesized in large quantities, with minimal potential pathogen infection [241, 242]. LMPs are often synthesized using commercial phospholipids without the inclusion of ApoB-100 proteins. Kim et al. synthesized an LMP with cholesteryl oleate and triolein as the core and cholesterol and DC-Cholesterol as the surface structure. Paclitaxel was loaded in LMP at the reconstitution stage [243]. However, LMP normally requires certain LDL receptor-binding domains or specific ligands modification for targeted delivery. For example, Nikanjam et al. synthesised LMP that possessed a lipid-binding motif and ApoB-100 LDLR binding motif to target glioblastoma with LDLR expression [241]. One key characterisctics of LMP is the ability to regulate LDLR expression. Dox has been loaded into LMPs modified with ApoB-100 and supplemented with mevastatin or simvastatin, resulting in the upregulation of LDLR expression [244].

A number of studies have demonstrated atheroprotective effects of LMPs. Synthetic cholesteryl-core LMPs loaded with paclitaxel administered to LDLR knockout mice on a 1% cholesterol diet resulted in reduced atherosclerotic lesions, including a 14% reduction in wall area and a 22% reduction in stenosis [245]. In a similar study, cholesteryl-core based LMPs loaded with paclitaxel, were injected into rabbits fed a high cholesterol diet. These LMPs showed significant atheroprotection, demonstrated by a reduction in intima, lesion extension and number of macrophages in the intima [246]. Carmustine has also been loaded into cholesteryl-core LMPs and reduced the lesion size by 90% and inhibited the accumulation of macrophages, T cells and vascular SMCs at the intima in a rabbit model [247].

**Figure 4.**
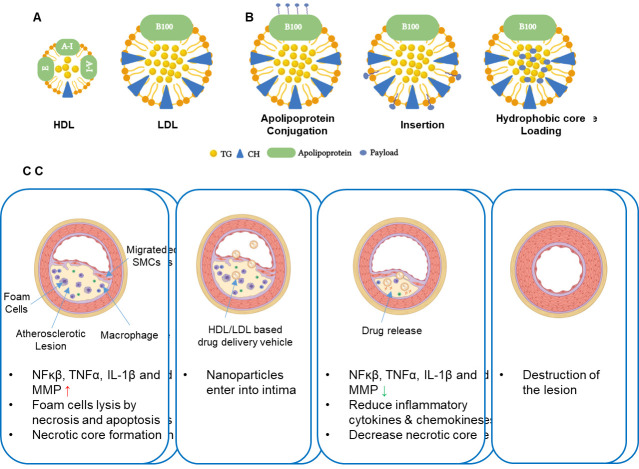
HDL and LDL based nano carriers. (A) the structure of HDL and LDL [252]. (B) Three strategies to fabricate rHDL [253]; and (C) HDL/LDL based nano carriers for atherosclerosis treatment [254]. All figures are recreated using Biorender.

Amphiphilic sugar-based molecules (AMs) have been used to synthesize ox-LMPs, which have similar charge and hydrophobicity to ox-LDL. AMs can bind with scavenger receptors. Tian et al. synthesized ox-LMP from amphiphilic polymers with alkyl chains on mucic acid binding to hydrophilic PEG chains, resulting in the self-assembly into ox-LMP [248]. The same group showed that one rotationally restricted carboxylic acid within AMs adequately inhibits THP-A human macrophage take up of ox-LDL [249]. Lewis et al. synthesized ox-LMPs using AM micelles and serum-stable PEG nanoparticles which bound with MSR1 and CD 36, which decreased ox-LDL accumulation and foam cell formation [250]. Petersen reported that AMs based nanoparticles downregulated the expression of scavenger receptors, SRA and CD 63, leading to the transformation of atherogenic macrophages to athero-resistant phenotype [251].

Taken together, the recent advances in HDL and LDL based drug delivery systems showcase the potential for these novel therapies to treat atherosclerosis (Fig. 4). Long-term cytotoxicity and therapeutic effects as well the clinically relevant dosing need to be fully evaluated in preclinical large animal models, before translation to clinical studies.

## 7. Conclusions and future perspective

Liproproteins remain an increasing focus of research due to their close association with atherosclerosis. Extensive studies have shown that apoB-containing lipoproteins facilitate the development of atherosclerosis. HDL generally displays anti-atherogenic properties, whilst dysfunctional HDL may promote the development of atherosclerosis. Studies highlighting novel diagnosis and other biomarker indications and treatment strategies targeting lipoprotein metabolism demonstrate increasing potential for clinical translation. However, key challenges remain. The growing understanding of heterogeneity of structure and function of lipoproteins has complicated elucidation of exact mechamisms underlying lipoprotein contributions to atherosclerosis. More work is required to develop precise and readily available diagnostic markers and to identify drugs with appropriately targeted efficacy. Most knowledge to date is based on *in vitro* cellular studies and animal models. Predicting which of the promising strategies explored to date will translate to the clinic is not yet clear and requires further investigation.
